# Supplementary material for: Are carbon emissions trading and green financial instruments synergistic? -Comprehensive quantitative research based on content analysis
Source: PLoS One. 2024 Mar 7;19(3):e0298601. doi: 10.1371/journal.pone.0298601 (PMC10919601; doi:10.1371/journal.pone.0298601)
Supplement: S1 Data — (ZIP) [file pone.0298601.s001.zip › Data/地级市Z_F工作报告.zip!/┴╔─■/╕o╦│╩╨/└┤╫╘╣1⁄2╓┌║┼í╛┬φ┐╦╩2╛▌═°í┐_2016.docx]

各位代表：

现在，我代表市人民政府，向大会作政府工作报告，请予审议，并请市政协委员提出意见。

一、“十二五”回顾及2015年工作

过去的五年，在省委、省政府和市委的正确领导下，在市人大和市政协的监督支持下，市政府带领全市人民，抢抓历史机遇，积极应对严峻挑战，全力以赴稳增长、促改革、调结构、惠民生，开创了城市转型振兴、幸福美丽抚顺建设的新局面。

五年来，我们坚持经济发展与民生改善并重，经济实力提升，人民生活水平提高。预计地区生产总值年均增长8.4%，城镇居民人均可支配收入年均增长11.9%，农村居民人均可支配收入年均增长12.9%。

五年来，我们积极推进沈抚同城化，与沈阳共享“024”区号、交通同城等取得实质性进展，各项工作走在沈阳经济区同城化前列。深入实施“两城两带一区、多点支撑、全域发展”战略，沈抚新城与沈阳一体化发展格局初步形成，石化新城化工及精细化工产业丰厚度不断提升，县区竟相发展，南环产业带、东部生态保护区加快建设，浑河景观带成为抚顺靓丽风景线。

五年来，我们着力优化存量、扩大增量，全面提升发展质量。抚顺特殊钢股份有限公司等骨干企业转型升级初见成效，千万吨炼油、百万吨乙烯等重大项目建成投产。到2015年末，服务业增加值占地区生产总值比重达到42%，比2010年提高6.8个百分点。

五年来，我们全面深化改革、扩大开放。完成省政府取消238项、下放38项行政职权的衔接工作，公布市、县区政府部门权责清单，工商登记、财税体制和农村等重点领域改革取得积极进展。中国水电建设集团、中国供销集团、广州恒大、深圳万科等国内知名央企民企进驻抚顺，罕王集团投资印尼红土镍矿产业园成为辽宁实施“走出去”战略重点项目，经济发展动力和活力增强。

五年来，我们坚持生态优先、建管并重，大力实施青山、绿水、蓝天、畅通、清洁、景观六大工程。抚顺荣获“国家森林城市”称号，大伙房水源保护区成为国家江河湖泊生态环境保护试点和生态文明先行示范区。城区跨河大桥、主要街路改造一新，抚顺结束了没有动车的历史。月牙岛生态公园、人民广场等建成投用，城市绿化、亮化、美化、净化、冬季无害化除雪和宜居乡村建设等工作走在全省前列。

五年来，我们千方百计增加投入，尽心竭力办好惠民实事。企业退休人员养老金、城乡低保救助人均标准年均增长分别为15.2%、14.2%和17.9%。养老、医疗保险体系覆盖城乡，新农合参保率达到99.83%，城镇登记失业率控制在4.06%以内。保障性安居工程惠及16.2万人。妇女小额贴息贷款在全省排名第一。新建改造城区中小学（幼儿园）近百所、市级医疗卫生机构5家，新建市图书馆、雷锋大剧院、青少年宫、殡仪馆等投入使用。农家书屋实现全覆盖。调整延伸公交线路，基本解决市民出行难问题。

五年来，抚顺经受住了新世纪以来最为不利的经济形势和“8-16”特大洪灾等严峻考验，地区综合实力不断提升，城市面貌发生巨大变化，人民群众拥有了更多的获得感和幸福感。全市人民用智慧和汗水，共同绘就了令人骄傲和自豪的“十二五”美丽画卷。

各位代表：刚刚过去的2015年，抚顺经济社会发展遇到的困难比预想的多、面临的压力比预想的大，取得的成绩实属不易。预计地区生产总值增长2.8%；一般公共预算收入完成预算的53.9%、同比下降45.1%．剔除不可比因素同比增长2%；城镇居民人均可支配收入增长7.1%；农村居民人均可支配收入增长7.2%。

1、经济保持平稳运行

支持企业发展。累计为企业减负1.94亿元，争取国家、省工业项目资金支持7.23亿元，促成企业间协作配套1.1亿元，抚顺石化公司等骨干企业支撑作用明显，全年规模以上工业总产值完成2077亿元。

主动抢抓机遇。细化落实《国务院关于近期支持东北振兴若干重大政策举措的意见》，争取中央预算内补助资金1.2亿元、资源枯竭型城市财力转移支付资金5.7亿元，争取国家开发银行等专项债券6.7亿元，新抚区老工业区搬迁改造完成投资8亿元。

加快项目建设。投资97.4亿元的清原抽水蓄能电站项目加快推进，投资45亿元的丰远·热高乐园基本建成，全年开复工亿元以上重点项目209个、完成投资314亿元。

强化金融支撑。全年新增本外币贷款98.5亿元、非信贷融资175.9亿元，小微企业贷款比年初增加45.7亿元，低利率为企业拆借“过桥”基金1.5亿元。联合上海股权交易中心，成立抚顺市企业挂牌孵化基地。1家企业实现新三板挂牌，2家企业报审主板上市。政府融资规模突破50亿元。

2、改革开放全面深化

重点领域改革成效明显。结合抚顺实际，自行取消27项、下放32项市级行政职权，建设工程项目审批时限由275天压缩到40天，沈抚新城在全省率先成立行政审批局。工商登记、财税体制、国资国企和农村改革等进展顺利。在全省率先开展民生领域证明材料专项清理。

资源型城市转型研究工作扎实推进。国务院发展研究中心提出，在抚顺等典型性、代表性、发展潜力较大的资源型城市，设立“国家资源型城市可持续发展转型创新试验区”的课题报告，得到国务院领导的关注和批示。抚顺在国内资源型城市中率先完成《国家资源型城市可持续发展转型创新试验区顶层设计及系统发展规划》，创建国家资源型城市可持续发展转型创新试验区得到省政府的重视和支持。

对外开放取得积极进展。成功举办辽宁百家知名民营企业抚顺招商投资洽谈暨名优企业名优产品展示活动。广州无线电集团金融服务外包产业园、韩国LEO株式会社与抚顺森源集团合作研发的电动车（船）等项目成功签约，实现金融和对韩招商新突破。全年引进域外资金209亿元，实现外贸出口6.5亿美元。

3、三次产业稳步发展

工业发展质量提高。传统产业完成技改投资145亿元，竣工投产项目75个。与抚顺石化公司、抚顺矿业集团战略合作取得积极进展。与大连理工大学、辽宁石油化工大学等深入合作，组建化工新材料、智能制造等产业技术创新联盟。辽宁三友生物科技公司等8家单位获批省级产业专业技术创新平台。高新技术产品增加值占规模以上工业增加值比重达30%。

现代服务业快速发展。抚顺被中国城市竞争力研究会评选为中国最具特色旅游城市，策划推出十大特色景区、十大精品线路等，全年接待游客总人数、旅游总收入分别增长l1%和l5%。加快电商平台建设，东北亚钢铁网、首华（辽宁）农产品交易中心等上线运营，全年电子商务交易额实现300亿元。海油集团金融大厦等重点金融项目加快建设，全年金融业实现税收9亿元。

特色农业规模不断扩大。新增有机农业4万亩，形成有机中药材、短梗刺五加等六大特色产业示范基地。食用菌、中药材、山野菜、特色精品花卉种植规模居全省前列。

4、区域经济协调发展

沈抚新城被确立为省级电子商务集聚示范区，千亿元休闲娱乐旅游集聚区初具雏形，与沈阳共同争取国家批复沈抚新区进展顺利。

石化新城高新技术产业园区公用工程一体化建设进展顺利，开工建设亿元以上工业项目13个，投资3亿元的森源化工项目当年建设当年投产，海赋外滩商业广场建成投用，新屯公园、河滩公园、紫花岭风景区完成提升改造。

抚顺、清原、新宾三县工业产业集群初具规模，抚顺县三块石国家森林公园被《香港商报》等媒体评为中国最佳旅游风景区，清原县成为国家级电子商务进农村综合示范县，新宾县获批省级农业标准化示范县。

新抚、顺城、望花三区错位发展，新抚中央大街地下水城、顺城泰为国际文化汽车城、望花小微企业创业示范基地等项目加陕建设。

5、城乡环境持续改善

环境保护力度加大。大伙房水源保护区综合治理取得初步成效，建成乡镇污水处理设施20个，一级保护区实现全封闭，生态移民、污染治理等工作进展顺利。大气污染综合整治深入推进，淘汰10吨及以下燃煤锅炉338台，对重点排污企业驻厂监督、限产限排，严禁城区焚烧落叶，集中整治建筑工地、露天堆场和街路扬尘，启动“青草计划”。持续推进青山工程，营造生态林3.25万亩，封山育林4.5万亩。

城市功能不断完善。《抚顺市城市总体规划（2011-2020）》经过部省联审。辽宁中部环线抚顺段高速公路建设稳步推进，新建改造“三供”管网163.4公里。城市导览导视系统建设有序推进，新建改造游园、绿地、广场100处，新增绿地面积32公顷。点亮万盏灯、照亮回家路的“居民小区照明计划”进展顺利。

宜居乡村建设扎实推进。维修改造农村公路197公里，完成147个行政村的村屯绿化工程，农村卫生厕所改造3720座，全市16个村被省政府列为美丽乡村示范村。

6、民生保障扎实有力

社会保障水平不断提升。企业退休人员养老金月人均调增201元，城乡低保提标7%和10%。大病保险人均筹资标准由18元提高到25元；人均基本公共卫生服务经费由35元提高到40元。低保对象基本医疗住院救助比例由60%提高到70%，重特大疾病救助比例由50%提高到60%。城镇居民医疗保险、新农合财政补助由每人320元提高到380元。18万符合条件困难企业退休人员纳入医保范畴。扶持创业带头人824人，创业培训2109人，新增城镇实名制就业7.5万人。

安居扶贫工作成效明显。完成1.5万户棚改任务，维修改造棚改房屋1295栋，实施“暖房子”工程50万平方米，改造农村危房610户、老旧房屋室内供水设施2万户。1.59万国家标准农村贫困人口稳定脱贫，“互助式扶贫”工作走在全省前列。

各项社会事业协调发展。推进教育均衡发展，10所幼儿园成为“辽宁省五星级幼儿园”，3个城区通过国家义务教育发展基本均衡县区验收，抚顺职业技术学院办学实力跃居全省同类院校前列。市中心医院、二院分别与医大一院和盛京医院深度合作，7家县级公立医院全部取消药品加成，农村县、乡、村三级医疗机构实现基本药物制度全覆盖。推进文化惠民工程，新建社区书屋141家，开展基层群众文化活动2200余场。抚顺“琥珀城”被授予国家级文化产业示范基地。加强体育设施场地建设，成功举办亚洲大学生龙舟锦标赛、全国山地自行车冠军赛和全国首届越野车场地赛等。市老干部大学拥有独立办学场所。

社会治理能力不断加强。完成309个社区换届工作，开工建设城市社区办公用房50个。信访总量下降15%，为近年最好水平。事故救援、抗灾减灾能力进一步提升，安全生产和食品药品安全形势总体稳定。刑事案件发案率下降5.6%，群众安全感增强。

7、政府自身建设不断加强

坚持依法行政。牢固树立法治观念，深化政务公开，完善决策程序，不断健全权力运行制约和监督体系，切实做到有权必有责、用权受监督，政府工作不断走上法制化、规范化轨道。

改进公共服务。开展换位体验活动，查找问题，改进和优化公共服务流程，公共服务质量和水平不断提高。

加强作风建设。深入开展“三严三实”专题教育，党员干部的服务意识、责任意识、担当意识、发展意识不断增强。

广泛接受监督。自觉接受市人大及其常委会的法律监督、工作监督和市政协的民主监督，主动听取民主党派、工商联和社会各界意见建议。全年办理人大代表建议328件、政协委员提案389件，办结率lOO%、满意率99%。

各位代表：回首过去的五年，我们在攻坚克难中奋力前行，在抢抓机遇中积极作为，迈出了城市转型振兴发展的新步伐。成绩的取得，是省委、省政府和市委正确领导，市人大、市政协大力支持和全市广大干部群众共同努力的结果。在此，我代表市人民政府，向全市各族人民，向人大代表、政协委员，向各民主党派、工商联、无党派人士、人民团体、离退休老同志，向驻抚部队、武警官兵、公安干警和中省直单位，向所有关心支持抚顺发展的海内外朋友们，表示崇高的敬意和衷心的感谢！

看到成绩的同时，我们也要直面问题。一是受宏观经济形势和做实经济数据等因素影响，“十二五”和2015年部分经济指标没有达到预期发展目标。二是传统产业亟待改造升级，新兴产业和接续产业尚未形成规模，产业、产品结构调整严重滞后。三是民营经济规模不大、活力不足，大众创业、万众创新积极性不高，“双创”氛围还不浓厚，严峻经济形势造成就业压力加大。四是部分区域空气异味严重，城市雾霾天气频发，大气污染治理任务艰巨。五是公共服务质量不高，少数政府工作人员慢作为、不作为、乱作为，担当精神、责任意识不强，干部士气亟待提振，经济社会发展环境亟需改善。

二、“十三五”时期的总体要求和目标任务

各位代表：“十三五”时期是抚顺转型振兴发展的攻坚阶段，也是全面建成小康社会的决胜阶段。我们依然面临着严峻复杂的国内外经济形势：从国际看，石油、铁矿石等大宗商品价格走低，市场持续动荡，经济复苏缓慢；从国内看，经济发展仍处于“三期”叠加阶段，经济增长新旧动能正在转换之中，产能过剩、投资增速放缓、金融风险上升加大了经济运行的困难；从全省看，长期积累的结构性、体制性问题不断凸显，经济增速回落明显；从抚顺看，抚顺作为国家老工业基地和资源枯竭型城市，长期积累的矿山地质灾害严重、采沉影响区域扩大、养老保险金缺口巨大、厂办大集体问题突出、财政收支困难、政府债务率偏高和人口长期流失等历史遗留问题与现实矛盾相互交织、并存叠加。同时，我们更要看到，党中央、国务院支持东北老工业基地振兴的决心和力度有增无减，抚顺发展面临国家进一步推进新一轮东北振兴、沈抚同城化、与沈阳共建沈抚新区，以及国家重视资源型城市转型发展、支持央企与地方共建产业园区和采煤沉陷区综合治理，国家和省积极推动大伙房水源保护等一系列重大机遇。此外，抚顺转型振兴的目标明确、战略明晰，拥有雄厚的产业基础、国内领先的科研院所和数量庞大的产业技术工人，具备大众创业、万众创新的有利条件。只要我们坚定信心、众志成城、奋发图强，再大的困难，也阻挡不了抚顺前进的脚步，动摇不了全市人民追求幸福、追逐梦想的决心。

“十三五”时期发展的总体要求：以党的十八大和十八届三中、四中、五中全会精神为指针，认真贯彻党中央关于东北振兴的最新重大决策部署、中央经济工作等会议精神，牢固树立创新、协调、绿色、开放、共享的发展理念，积极适应和把握经济发展新常态，以提高发展质量和效益为中心，以城市转型振兴为主线，深入贯彻“四个着力”要求，坚持变中求新、变中求进、变中突破的工作总基调，加大供给侧结构性改革力度，保持战略定力，增强发展自信，全力推动去产能、去库存、去杠杆、降成本、补短板五大任务，深入实施“两城两带一区、多点支撑、全域发展”战略，积极构建科学精细的经济社会管理模式，创造宜居乐业的经济社会发展环境，努力提升发展活力、内生动力和整体竞争力，实现更有质量、更有效率、更加公平、更可持续的发展。

“十三五”时期发展的重点任务：一要着力完善体制机制。转变政府职能，强化服务意识，全力推进供给侧结构性改革，充分发挥市场在资源配置中的决定性作用。二要着力推进结构调整。改造提升传统优势产业，培育壮大战略性新兴产业和现代服务业，大力发展以本地产品为原料的接续产业，加快形成绿色低碳高效的产业发展支撑体系，改变传统产品占大头、“原”字号“初”字号产品居多的单一产品结构，提高消费对经济增长的贡献率。三要着力鼓励创新创业。积极构建有利创新创业的政策和制度环境，深化“政产学研金用”合作，加快形成“双创”新局面。四要着力保障改善民生。持续加大民生投入，大力发展社会事业，全面改善生态环境，不断增强全市人民的获得感和幸福感。

五年后的抚顺，重点领域和关键环节改革有重大突破，转方式调结构取得实质性进展，地区生产总值和城乡居民人均收入比2010年翻一番，地区综合实力全省排名前移，国家标准农村贫困人口实现稳定脱贫。新型城镇化进程加快，城乡协调发展、空间布局更加科学合理，城市综合承载能力不断增强。生态环境质量明显改善，主体功能区和生态安全屏障基本形成。依法治市体制机制更加完善，公共服务效能全面提升，社会治理体系和治理能力现代化取得重大进展。社会更加公平正义、诚实守信，社会主义核心价值观深入人心，雷锋精神进一步弘扬，抚顺人对家乡的认同感、归属感和自豪感明显增强，全市人民共同步入小康社会。

二、2016年重点工作任务

2016年全市经济社会发展的主要预期目标：地区生产总值增长5%以上，一般公共预算收入增长2%，城乡居民收入增长高于经济发展水平，民生、社会事业、环保投入高于上年水平，单位生产总值能耗下降2%，主要污染物排放量削减率完成省下达目标，经济发展质量和效益进一步提高。

今年要重点做好以下工作。

1、继续强化工业支撑

围绕《中国制造2025辽宁行动纲要》，全面实施抚顺智能制造2016-2018行动计划，重点实施100项智能制造技术改造项目，推进30个智能制造企业示范工程，推动传统低端制造向中高端制造升级。

改造提升传统产业。坚持创新驱动，积极引进先进科技成果和技术工艺，加快推动抚顺石化公司扩大“油蜡联产”产能和恢复乙烯化工厂生产，推进抚顺特殊钢股份有限公司特冶技改二期等重大技改工程，促进石化、冶金等传统产业绿色改造，向智能化转型升级。全面提倡、推动企业优化管理，降低成本，提高效率。积极稳妥化解过剩产能、处置“僵尸企业”。

加快发展新兴和接续产业。准确把握国家政策导向，发展壮大新材料、新能源汽车、节能环保和电子信息等战略性新兴产业，全力打造机器人产业配套基地。利用抚顺石化、特钢等产品资源，延伸加工，拉长产业链，拓宽产业丰厚度，增加终端产品比例，积极培育新的经济增长点。

提升企业自主创新能力。实施科技型中小企业成长、高新技术企业倍增、知识产权优势企业培育等计划，引导重点产业建立技术创新联盟，积极发展高新技术产业。鼓励、支持企业自主研发核心技术、引进先进技术、开发新产品，发挥科技创新对工业的支撑作用。

2、着力抓好项目建设

科学精细策划包装项目。围绕国家提出的”7+4”工程包和十大重点领域，各县区、开发区和相关部门要密切跟踪政策导向，列出对上争取项目计划清单，明确责任抓落实。围绕全市产业基础、转型方向和功能定位，积极策划一批对外招商合作的产业和社会事业发展项目。依托现有存量城市公共基础设施资源，策划包装和推进实施一批PPP模式融资项目。

促进一批重点项目尽快开工建设、投产达效。扎实做好三宝屯污水处理厂中水回用等项目开工前准备工作，加快推进抚顺矿业集团热电厂等项目建设，全力争取清原抽水蓄能电站项目下半年开工建设。全年开工建设亿元以上重点项目209个、完成投资350亿元。

集约高效盘活利用现有要素资源。全面摸清全市闲置土地、厂房等情况，对长期未开工建设、竣工投产的项目，促其引进新项目或转产；对长期闲置资源依法依规收回并盘活。合理调整产业雷同、基础薄弱的工业园区，深入挖掘现有要素资源潜力，集中对外招商、推进项目建设。

3、持续改善公共服务

集中审批。具有行政审批职能的政府部门全部成立审批处，审批职能全部划入审批处，统一进入公共行政服务中心集中审批，实行“一站式”服务，切实做到“中心之外无审批”。进一步明确公共行政服务中心功能定位，加快功能升级，承担起全面改进公共服务的推进和监督职能。

下放权力。继续取消和下放一批市级行政职权事项，将各种涉及百姓和企业的公共服务事项向基层下沉，最大限度便民利企。

强化服务。大力推行网上审批、并联审批，优化建设项目审批流程。清理规范行政审批中介服务，取消各类不合法、不合规的收费和证明。分别在市发改委、科技局、经信委设立日常协调机构，建立由市发改委牵头的联席会议制度，及时协调解决驻抚中省直单位、科研院所、大专院校、民营企业日常运行和发展中遇到的困难与问题。

换位体验。具有公共服务职能的机关和事业单位，持续开展换位体验活动，继续改进和优化公共服务流程，让群众和企业办事顺心、生活舒心、投资放心。

4、深化重点领域和关键环节改革

深化商事制度改革，巩固扩大“三证合一”、“一照一码”改革成果，进一步简化工商注册资本登记，推进工商注册制度便利化，落实企业信用信息公示。

深化国资国企改革，健全公司法人治理结构，建立市场化用工用人机制，提高国有资本经营质量效益，做强做优国有企业。抓好国有资本运营、投资公司创建和运营，推进经营性国有资本集中统一监管，整合盘活国有资产存量。

落实国家和省财税体制改革部署，优化政府预算结构，推进政府预决算信息公开；全面推进营改增改革，加快资源税改革；优化政府债务结构，加强债务管理，积极推进政府债务化解和风险防范工作。

深化农村改革，加快农村集体土地承包经营权确权登记颁证工作，大力培育新型农业经营主体，引导土地承包经营权规范有序流转，发展农业适度规模经营。

加快进环保、医疗、公用事业等重点领域价格改革。推进公务用车改革和机关事业单位养老保险制度改革。

5、全力推进创新试验区建设

抓住新一轮东北振兴重大机遇，全面梳理抚顺作为东北老工业基地城市为国家作出的贡献，以及历史遗留问题对现实发展的影响，全力争取国家政策支持，积极创建抚顺资源型城市可持续发展转型创新试验区，探索体制机制创新、矿城共融共兴、以建促治促转、产业多元推动的转型发展模式，逐步从根本上解决矿山地质灾害区生态恢复、接续产业发展和居民安居就业等问题。

大力推进东北商贸城、森茂国际汽车城、创意文化产业园区，大型商务会展产业园区、中国北方汽车文化主题产业园、生态运动廊道、建筑设计博览园等项目建设。加快儿童运动公园及跨东林路人行景观桥建设，建成创新试验区规划展示馆、国家区域性（抚顺）地理国情监测中心、采沉记忆实景公园。

6、全面提升开放水平

进一步强化开放意识。选择国内外先进地区、发达城市，比对思想观念、工作方法和各项指标，充分借鉴成功经验，见贤思齐，比学赶超，加快推进开放型经济体系建设。全面开展与重点商协会合作，积极营造全方位扩大开放的深厚氛围。

狠抓招商引资。坚持招商引资与招才引进智相结合，瞄准长三角、珠三角、京津和海外地区，注重行业招商、专业招商、精准招商、以商招商，积极开展小团组、点对点、面对面招商，着力引进一批重大项目、先进技术和优秀人才。

扩大外贸出口。深化与中国航天长征国际贸易公司等军贸企业合作，培育服务外包等新兴业态，加强出口基地建设。启动与中国对外承包工程商会合作，研究组建抚顺市海外工程商会，带动劳务、技术、装备和产品输出。

7、积极鼓励创新创业

加大宣传引导力度，营造大众创业、万众创新的浓厚氛围。广泛调动社会各界力量，鼓励各级各类科技人才、各行各业技能人员和普通劳动者，开展多种形式的创新创业活动。全面总结推广抚顺特殊钢股份有限公司、沈阳隆基电磁科技股份有限公司创新创业的成功经验，发挥先进企业的示范引领作用。

全面落实国家和省推进大众创业、万众创新政策措施。设立创业投资（引导）基金，支持创新创业，鼓励小改小革、点滴创新，调动“双创”积极性。加快总部基地创投中心、东北振兴投融资项目网络平台等“众创”载体建设，推动“互联网+”融合创新。立足抚顺科技和产业优势，联合企业、金融、保险等单位和机构，建立科技创新联盟，加快“1+3+N“能源化工孵化器建设，促进科技成果加速转化和产业化，壮大抚顺相关产业。

深化与抚顺石化公司、抚顺矿业集团等中省直大企业合作，促进地企共兴共荣。深化与驻抚科研院所、大专院校合作，加快形成以企业为主体、市场为导向、“政产学研金用”相结合、全社会共同参与的科技创新体系。加强与军工企业合作，鼓励“军转民”，支持“民参军”，促进军民科研成果和制造技术双向转化。加强与其他驻抚中省直单位合作，力争在行业引导、产业发展、项目建设等方面取得更大成绩。

8、大力发展民营经济

营造良好发展环境。全面改善提升服务水平，大力整治推诿扯皮、吃拿卡要、办事拖沓等现象，打造公平开放的投资环境、竞争有序的经营环境、体贴到位的服务环境。对企业一视同仁，保障企业公平参与市场竞争、平等使用生产要素、同等受到法律保护，努力做到规则公平、机会均等。

鼓励扶持企业发展。全面落实各项鼓励扶持政策，主动帮助企业争取政策资金支持，降低企业生产经营成本。依托海油集团金融大厦，建成集信贷、评估、担保于一体的中小企业金融服务中心。用好产业投资（引导）基金和中小企业“过桥”基金，鼓励中小企业进入辽宁股权交易市场挂牌交易，支持企业上市，解决企业“融资难”、“融资贵”问题。

大力倡导和培育企业家精神。厚植民营企业发展土壤，建立健全激励和约束机制，营造企业家成长的良好环境。大力倡导做精主业、开创新业、追求卓越、诚实守信、回馈社会的企业家精神，让企业家精神成为抚顺产业结构调整、城市转型发展的强大动力，充分发挥企业家引领产业革新、推动经济发展的主力军作用。

9、加快发展服务业

大力发展旅游业。树立大旅游观，深入挖掘历史文化、风俗民情、工业文明内涵，包装、策划、建设具有地域特色的旅游项目，推动旅游与文化、体育、工业、农业等产业深度融合。

大力发展乡村、少数民族村寨、沟域、森林旅游，建设大伙房水库周边生态旅游区。推进沈抚旅游一体化发展，建设沈阳经济区生态休闲旅游集聚区。全面整合旅游资源，组建旅游集团。加大宣传营销力度，积极推进智慧旅游建设，打造具有市场竞争力的旅游品牌，策划推出“多日游”，建设旅游目的地城市，充分发挥旅游对服务业发展的牵动作用。

改造提升传统商贸业。创新业态和经营模式，坚持“线上线下”互动，促进传统商贸业转型升级。科学合理规划商业集聚区，推进南站中央商务区和北站新华商圈建设。

加快发展电子商务。加快同城物联网大厦、“互联网小镇”等电商平台建设，推进清原电子商务进农村综合试点县、沈抚新城省级电子商务集聚示范区建设，促进网络经济与实体经济融合发展。

大力发展生产性服务业。加快现代商品流通体系建设，完善物流配送网络，研究组建包装、配送、快递等服务公司，打造一批以本地特色农产品、钢铁、琥珀等为支撑的全国性和地域性商品网络交易中心。

加快培育现代金融体系。加强统筹协调，进一步壮大金融体量，用好资本市场，拓宽融资渠道，完善金融体系，大力发展产业金融。促进融资租赁、投资基金等多元化主体发展，积极筹建融资担保集团。推进沈抚新城和新华金融集聚区建设。加快农村信用社改制，促进小微金融、农村金融、互联网金融发展，发挥小贷公司、担保公司作用。

积极消化房地产库存。鼓励房地产开发企业顺应市场规律调整营销策略，适当调整商品房价格，薄利多销。大力推行棚户区、旧城区改造货币化安置，将去库存与新型城镇化、棚改房、公租房、拆迁安置房相结合。用好网交会、房交会等平台，全力消化存量商品房。科学调控房地产开发速度与规模，促进房地产业健康发展。

10、加快推进两座新城建设

沈抚新城，要加快融入沈阳经济区，积极与沈阳共同争取国家批复设立沈抚新区。重点推进与沈阳互联互通基础设施建设、互补错位产业项目建设，积极争取获批国家应急产业基地，引入沈阳更多的教育、医疗、文化等优质公共服务资源，与沈阳分享更多优质公共服务。

石化新城，要加快与央企共建产业园区，重点推进供热、管廊、消防、危化品物流等基础设施项目共建共享。充分利用抚顺石化公司原料资源，大力发展化工及精细化工产业，拉长产业链，提高原料加工深度和产品附加值。

11、扎实做好农业农村工作

实施品牌战略。整合优势农产品资源，培育、引进一批知名农业产业化龙头企业，研究建立面向沈阳市场的特色农产品配送中心，逐步形成具有一定规模和影响的加工输出基地，打造抚顺特色品牌，带动农民增收致富。

发展特色农业。依托大伙房水源保护区绿色、有机农业示范基地建设，加快推进“三品一标”认证工作，完成绿色、有机食品认证面积20万亩。继续扩大食用菌、中药材等特色产业基地规模，做强做大农产品交易中心。

壮大县域经济。坚持生态立县、绿色发展，做大抚顺县木制品加工、清原县特种输变电、新宾县现代焊接新材料等工业产业集群，大力发展农产品深加工业和特色旅游业，富民强县。

加强农业基础设施建设。加快实施灌区续建配套和节水改造工程，新增节水灌溉面积1万亩、高标准基本农田1.48万亩。新增农机动力3万千瓦，主要农作物综合机械化水平达到75%。

继续推进宜居乡村建设。大力实施农村环境综合整治，推进垃圾分类处理、畜禽粪便集中处理，提升污水处理技术水平，改善农村能源结构，做好农村改厕和农药、化肥包装等废弃物的回收治理工作，逐步全面实现村路硬化、村庄亮化、村容美化、村屯绿化，加快美丽乡村建设。

12、进一步做好城市工作

提升规划水平。综合考虑城市功能定位、文化特色和地域环境等因素，从地下、地面和空间三个层面，对城市进行立体、长远和协调规划，赋予城市更多的民族、文化、历史元素。加快编制城市地下综合管廊、海绵城市建设等专项规划，推动城市发展由外延扩张式向内涵提升式转变。

加强基础设施建设。多渠道筹措资金，加快辽宁中部环线抚顺段高速公路建设，规划启动安城桥建设。推进“三供”管网绿色低碳化改造。加快詹家河、鲍家河绿化升级改造和劳动公园动物园整体改造。启动建设商业设施互联互通工程，让南站、新华地区商圈更加畅通便民。全面维修改造中央大街、新抚路、章党街等街路。安装南环产业带碾三线路灯。

将有限财力向与民生密切相关的小街小巷倾斜，建设街头绿地和健身广场50处，安装居民小区照明灯具1.1万盏，让居民休闲健身有场地、夜晚回家有灯光。

加强城市精细化管理。健全城市治理体系，让广大市民参与城市管理，提高城市治理能力和执法工作水平。加快城市管理信息资源整合，推进“智慧城市”建设，发展民生服务智慧应用。建设、规范和改造中心城区停车场设施，新建公共厕所10座，设置垃圾桶、果皮箱各2000个。加强市政设施维修养护，科学设置导览导视系统和城市交通指示牌，大力整治“小凉快”、占道经营和私搭乱建，加强物业和弃管小区管理，倡导文明祭祀，改善提升市容环境。

13、加快推进新型城镇化建设

积极推进以人为核心的新型城镇化。科学规划、合理布局，以城带乡、城乡共建，重点发展县城和小城镇，着力破除城乡二元结构，推动产城融合发展。协调推进城镇化和新农村建设，实现城区辖乡镇、村庄规划全覆盖。遵循规律、因势利导，有序推进农业转移人口市民化，逐步实现城镇基本公共服务常住人口全覆盖，切实维护农民合法权益。

保护历史文化遗产。编制历史文化名城保护规划，正确处理文化遗产保护与新型城镇化建设和旅游开发的关系，坚持保护为主、适度合理开发，严守历史文物保护红线，防止建设性破坏，禁止破坏性开发，绝不以破坏历史文化遗产为代价，谋求一时的经济发展。

14、全面改善生态环境

加强水源保护和水污染治理。继续抓好大伙房水源保护区综合治理，启动一级保护区内商业网点搬迁工作，加快苏子河河口湿地恢复等一批重点治理项目建设，争取国家和省尽快建立完善资源有偿使用和生态补偿制度。严格实施水污染防治行动计划，不断提高浑河流域水体质量。

深入实施大气污染综合整治。以防治雾霾为重点，拆除城区10吨以下燃煤锅炉423台；治理城市建筑工地扬尘、道路遗撒和露天烧烤；推进农村秸秆综合利用，严控秸秆焚烧；实施裸地不见天、青草全覆盖的“青草计划”，让城区裸露地面全部绿起来，全面降低扬尘。以消除空气异味为重点，加快化工等重点污染企业提标改造，大力整治榆林采沉区垃圾焚烧、小作坊小工厂污染和龚家沟地板城严重污染，不断改善空气质量。

继续提升森林生态环境建设水平。植树造林9.9万亩，封山育林1万亩。坚决打击乱砍乱伐、毁林造田等违法行为，保护好环城林带。

严肃追究破坏生态环境责任。加大环境执法力度，按时完成清理整顿环保违规建设项目工作，严肃惩处违法违规行为，让破坏生态环境的任性行为付出沉重代价。

各位代表，日益严重的雾霾，是大自然向我们发出的无声警告。我们必须牢固树立绿色发展、绿色消费理念，从政府做起，从每个企业、每个人做起，用心保护好我们赖以生存的环境，让抚顺天蓝水净、地绿山青。

15、大力发展社会事业

坚持教育优先发展。以促进公平、提高质量为核心，大力发展公办幼儿园，构建公益性、普惠性学前教育体系。继续推进义务教育学校校长、教师交流轮岗，促进义务教育均衡发展。推进高中教育优质特色发展，加快发展现代职业教育。努力培养一流校长、一流教师。继续做好语言文字工作。

提高医疗服务水平。着力做好疾病预防控制工作。推进公立医院综合改革，完善基本医疗服务体系，提高基层卫生计生服务能力。逐步推行“分级诊疗”制度，推动中医“治未病”服务。完善计划生育综合服务体系，落实“全面二孩”政策。

建设文化强市。大力弘扬地域文化、雷锋精神，对外树立形象，对内凝聚人心。加快市博物馆、群众文化中心、工人文化宫建设和整合，维修改造市老干部大学教学楼，开展丰富多彩的群众性文化活动。依托琥珀、煤精、旗袍、根艺等特色资源，大力发展特色文化产业。利用城东闲置待售的非住宅资源，建设文化创意及智慧产业集聚区。

发展体育事业。加快健身公园、广场和户外体育运动中心建设，逐步免费开放公共文体设施，深入开展全民健身活动。办好全国山地自行车冠军赛、三块石国际登山比赛等大型体育赛事活动。

大力推进社会养老服务机构建设，积极探索建立以家庭为核心、以社区为依托、以专业化服务为主的居家养老服务体系，推动医养结合，不断提高养老服务水平。

16、全力保障和改善民生

提高社会保障水平。继续强化养老保险扩面征缴，研究建立养老保险风险基金，确保离退休人员养老金按时足额发放。继续提高城乡居民最低生活保障和农村五保供养标准。完善社会救助体系，开展慈善救助活动，推进医疗救助“一站式”结算。做好残疾人工作。

实施就业安居工程。强化职业技能培训，落实“稳岗补贴”政策，重点支持高校毕业生就业创业，保障困难群体人员就业，城镇登记失业率控制在5%以内。加强保障性安居工程建设，新增棚户区改造6010户。实施“暖房子”工程200万平方米，完成老旧房屋室内供水设施改造2万户。

加大扶贫攻坚力度。坚持精准扶贫、精准脱贫，继续实行干部驻村帮扶和对口定点扶贫，确保1.5万国家标准农村贫困人口脱贫、60个省定贫困村销号。

切实做好民族宗教工作，促进民族团结进步。

虽然当前经济下行压力很大，财政收支矛盾日益凸显，但越是困难时候，我们越要高度关注民生、积极改善民生，应该为百姓花的钱一分都不能少花，能够为百姓办的实事一件都不能少办。政府将不遗余力惠民生，要让全市人民生活越来越好、幸福指数越来越高。

17、推进社会治理精细化

强化社会治理。加强社区基础设施建设，新改扩建城市社区服务站25个。加大村务公开力度，实现村务公开规范化、制度化、经常化。加快房屋产权交易中心搬迁。推进气象现代化建设。提高立体化社会治安防控水平，争创“社会综合治理平安市”。全面落实优抚安置政策，争创“双拥模范城”。

维护公平正义。畅通群众诉求渠道，各级各类公共服务机构要积极回应百姓合理诉求。完善劳动争议专业化预防和医疗纠纷第三方调解机制。保障农民工合法权益。做好法律援助工作。逐步将信访纳入法治化轨道，注重从源头上解决信访问题。

确保安全稳定。强化监管，切实保障食品药品安全。严格落实安全生产责任制，全面加强质量技术监督、行业监管、隐患排查、专项整治，高度关注西露天矿滑坡问题，严防重特大事故发生。做好防汛工作，加强中小河道治理，保证汛期安全。全面完成易燃易爆危化品企业厂区装置、工艺管线、储运设施等重大危险源分布图绘制和报备工作，消防、安监、环保等部门要完善实用的应急救援方案和应急资源指挥调度图册，提高应急处置能力。做好森林防火工作，保证森林资源和生态安全。强化公共安全风险防范意识，加强预判预警预防工作，确保城市安全。

18、启动一批重大课题研究

围绕事关全局、影响长远的重大问题，设立专题，借鉴国内外先进地区经验，组织专业人员研究或面向社会公开招标，集思广益，为政府决策提供科学依据。

启动加快建设智慧城市、推动资源枯竭型城市转型、弘扬地域文化促进城市建设发展等重大课题研究。

启动人才强市计划研究，吸引和留住人才。

启动高铁、轻轨、地铁、铁路等轨道交通项目建设的可行性和相互协调性研究。

启动会展业发展可行性研究，促进商贸、旅游、会展“三位一体”融合发展，提升城市知名度和影响力。

启动全民学习计划研究，提高市民文化素质、专业技能，培养健康向上的情趣爱好，使社会主义核心价值观更加深入人心。

启动扩大雷锋基金会影响力研究，全面弘扬雷锋精神，提升城市感召力和吸引力。

19、全面加强政府自身建设

严格按照法定权限和程序行使权力，建设法治政府。自觉接受市人大及其常委会法律监督和工作监督，主动接受市政协民主监督，广泛接受舆论监督、公众监督。

认真执行中央八项规定，严格落实党风廉政建设主体责任和监督责任，加强审计监察，坚决查处各类违规违纪行为。

全面倡导和推进科学精细的思想方法和工作方法，积极构建科学精细的经济社会管理模式，遵循客观规律、遵守法律法规、尊重发展实际，工作精益求精、细致入微、久久为功。坚决反对“忽悠”、语焉不详的不良作风，下大力气改进文风和会风，报告工作、拟定文稿等，要实事求是、言简意赅、精准陈述。

带头重信守诺、务实高效，以政府诚信带动社会诚信，以政府高效带动社会高效。

加强督查考核，抓铁有痕、踏石留印，履职尽责，敢担当，善作为，定一件、干一件、成一件，坚决整治执行不力、为官不为等问题。

以全市人民的共同事业凝聚人心、提振信心、鼓舞斗志，保持昂扬向上、奋发有为的精神状态，使广大公职人员在加快城市转型振兴、造福全市人民的伟大进程中，丰富生命历程、体现人生价值。

各位代表，今天的抚顺，正处在转型振兴发展的关键节点上，机遇与挑战并存，困难与希望同在。我们将同全市人民一道，在市委的正确领导下，以坚韧不拔之志、开拓创新之举，重铸抚顺昔日辉煌，共创抚顺美好未来！
